# Suppressing Cooperative Water Condensation through Nucleation-Controlled HIPF Architectures

**DOI:** 10.1021/acs.langmuir.6c03562

**Published:** 2026-09-17

**Authors:** Marek Wiśniewski

**Affiliations:** † Department of Materials Chemistry, Adsorption and Catalysis, Faculty of Chemistry, Nicolaus Copernicus University in Toruń, Gagarina 7, 87-100 Toruń, Poland; ‡ Institute of Advanced Studies, Nicolaus Copernicus University in Toruń, Wileńska 4, 87-100 Toruń, Poland

## Abstract

Hydrogen-bonded ionic-pair frameworks (HIPFs) provide a versatile platform for controlling interfacial hydration through molecular organization. Here, two crystalline melamine–trimesic acid (ME–TMA) HIPFs structures with fiber-like and plate-like morphologies, denoted F-HIPF and P-HIPF, were obtained by varying the nucleation conditions. Both materials exhibit low N_2_-accessible surface areas of 7.5 and 2.5 m^2^ g^–1^, respectively, providing a low-porosity platform in which hydration behavior can be separated from extensive permanent-pore filling. F-HIPF shows smooth, nearly reversible water uptake, whereas P-HIPF develops pronounced high-humidity hysteresis associated with more cooperative water retention. Direct, water-vapor adsorption and desorption, calorimetry reveals distinct loading-dependent enthalpic and entropic signatures. Operando difference infrared spectroscopy further demonstrates reversible hydration and different temperature dependences of the normalized initial response. Rate-derived kinetic descriptors indicate an enthalpically favorable but entropically penalized response for F-HIPF and an almost athermal, kinetically entropy-favored response for P-HIPF. When deposited on oxidized carbon fibers, F-HIPF increases irradiation-induced evaporation relative to bare CF, showing that a developed permanent pore network is not required for the observed enhancement.

## Graphical abstract

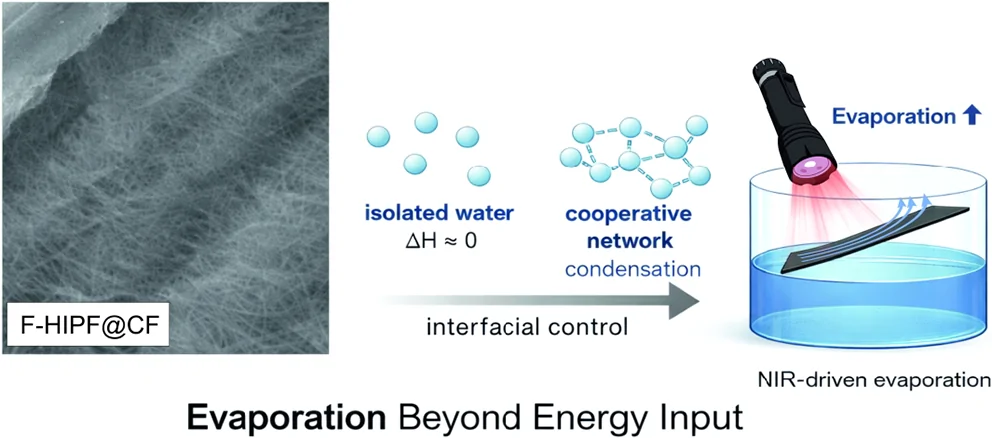

## 1 Introduction

Hydrogen-bonded organic frameworks (HOFs) and hydrogen-bonded ionic-pair frameworks (HIPFs) are supramolecular solids formed through directional hydrogen bonding, with HIPFs additionally involving ion-pair interactions between organic components.^1^ Permanent pore networks are central to many adsorption and storage applications of nanoporous solids,^2^ while HOFs and related shape-persistent organic architectures provide adaptable routes to organized molecular materials.^3,4^ Their reversible noncovalent interactions enable mild synthesis and structural adaptability, but also permit competing packing motifs during nucleation. Consequently, the same molecular precursors may form different crystalline phases when temperature, mixing, or nucleation history is changed.

Water adsorption is particularly sensitive to such differences in molecular packing. In porous and finely divided solids, uptake may progress from binding at favorable sites to the formation of increasingly correlated water populations. At high water activity, cooperative hydrogen bonding can produce steep uptake, hysteresis, and condensation-like retention in intrinsic pores, interlamellar spaces, or secondary voids between particles.^5–9^ Hysteresis alone, however, does not uniquely establish permanent porosity or equilibrium capillary condensation; equilibration kinetics and independent pore characterization must also be considered.

Infrared spectroscopy provides complementary information on the local hydrogen-bonding environment. Changes in the position, width, and shape of the O–H stretching envelope can reveal shifts in the distribution of water environments, although IR spectra do not uniquely determine cluster size or prove that individual molecules are isolated.^10–15^ A nearly invariant band position during uptake is therefore most safely interpreted as water occupying spectroscopically similar local environments, with limited evidence for progressive strengthening of a collective hydrogen-bond network.

Recent studies demonstrate that molecular organization can modulate humidity-dependent behavior even in systems without large permanent pore volumes.^16,17^ Metalloporphyrin-based humidity sensors provide a related example in which the central metal, solvent environment, and water coordination jointly alter the response to hydration, emphasizing the sensitivity of water–surface interactions to local molecular structure.^18^ These observations motivate the study of hydration as a property of the complete supramolecular interface rather than of surface area alone.

Here, melamine and trimesic acid were crystallized under two nucleation protocols to produce fiber-like and plate-like HIPF phases, denoted F-HIPF and P-HIPF, respectively. The F and P prefixes describe the observed morphologies without assigning one- or two-dimensional framework topology. SEM, PXRD, IR spectroscopy, N_2_ adsorption, water-vapor adsorption, direct adsorption calorimetry, and time-resolved operando IR spectroscopy were combined to compare the materials. F-HIPF was additionally deposited on oxidized carbon fibers (F-HIPF@CF) to examine whether its hydration response influences evaporation under near-infrared irradiation. The analysis separates equilibrium hydration thermodynamics from effective descriptors derived from the initial IR response. Related studies of water uptake, wetting, condensation, and liquid management at structured interfaces further show that macroscopic water transport depends strongly on interfacial architecture.^19–23^

## 2 Materials and Methods

### 2.1 Materials Synthesis

All chemicals were purchased from Sigma-Aldrich and used as received. Equimolar amounts of melamine (ME) and trimesic acid (TMA), 1 mmol each, were dissolved separately in 100 mL of deionized water. Each solution was stirred at 90 °C for 30 min.

For F-HIPF, the two hot solutions were combined under vigorous stirring for 1 min and cooled to room temperature at approximately 0.25 °C min^–1^ under thermally insulated conditions. For P-HIPF, the solutions were first cooled to 25 °C and then mixed, producing more rapid precipitation. The resulting solids were filtered and dried under vacuum at 25 °C.

The F-HIPF@CF composite was prepared by alternating immersion of oxidized carbon fibers in the melamine and TMA solutions. Four deposition cycles were performed, followed by washing and vacuum drying.

### 2.2 Structural and Spectroscopic Characterization

FTIR spectra were collected with a Nicolet iS50 FTIR spectrometer over 600–8000 cm^–1^. Difference spectra were referenced to the sample’s initial state. Thirty-two scans were accumulated at a resolution of 4 cm^–1^, and time-resolved spectra were collected at 30 s intervals.^24–26^

PXRD patterns were recorded using Philips X’Pert Pro θ–2θ and Bruker D8 diffractometers with Cu Kα radiation. Data were collected over 5–120° 2θ with a step size of 0.0084° 2θ. PXRD was used to establish the formation of two crystalline HIPF phases distinct from the parent compounds. The F and P designations were assigned independently of the observed SEM morphologies.

SEM images were obtained with a Quanta 3D FEG microscope. Samples were mounted on carbon tabs attached to aluminum stubs and analyzed without conductive coating.

### 2.3 Gas and Water-Vapor Adsorption and Calorimetry

The low temperature N_2_ adsorption–desorption isotherms were recorded at 77 K. BET analysis yielded apparent surface areas of approximately 7.5 m^2^ g^–1^ for F-HIPF and 2.5 m^2^ g^–1^ for P-HIPF. These low values provide a useful low-porosity platform for separating hydration and evaporation behavior from extensive permanent-pore filling. The full isotherms and analysis are provided in the Supporting Information (Figure S1).

Water-vapor adsorption–desorption isotherms were measured gravimetrically using an apparatus equipped with Baratron pressure transducers. Samples were evacuated at 50 °C until a constant mass was obtained. The principal isotherms were recorded at 25 °C. At each pressure step, pressure was monitored continuously, and equilibration was maintained for at least 5 h.

Water adsorption and desorption heats were measured directly using a differential Tian–Calvet-type adsorption calorimeter equipped with two geometrically identical cells operated as sample and reference channels.^27–29^ The differential configuration largely compensated for common thermal effects associated with gas expansion or compression, gas transport, and pressure-step perturbations. The remaining differential heat-flow signal was integrated and normalized to the amount of water adsorbed or desorbed during the corresponding step. Directional molar enthalpy changes were reported using the convention Δ*H*_ads_ < 0, for exothermic adsorption and Δ*H*_des_ > 0, for endothermic desorption.

The relative Gibbs free energy imposed by the water vapor was calculated as (eq 1):

1 $$\Delta G_{w}=\mathrm{RT}\;\ln\left(p/p_{s}\right)$$

where *p*/*p*_*s*_ is the relative water-vapor pressure and the reference is saturated water vapor at the same temperature. Mapping Δμ_w_ against the normalized uptake *q*/*q*_max_ shows the water chemical potential required by each material to reach a given fractional loading.

The corresponding direction-dependent entropy changes were calculated using a consistent process convention (eq 2):

2 $$\Delta S_{ads}=\frac{\Delta H_{ads}-\Delta G_{w}}{T},\Delta S_{des}=\frac{\Delta H_{des}+\Delta G_{w}}{T}$$

Thus, the exothermic adsorption and endothermic desorption signals retain their experimentally observed signs, whereas Δ*G*_w_ in Figure 2B remains the equilibrium water chemical potential relative to saturation for both branches.

### 2.4 Initial-Rate Analysis of Operando IR Data

The intensity of the difference-spectrum response was converted to a normalized progress variable, α­(*t*), ranging from 0 to 1. Because the initial difference spectrum corresponds to *A*_0_ = 0, the adsorption response was normalized as α_ads_(*t*) = *A*(*t*)/*A*_∞_. The desorption response was direction-adjusted so that it also increased from 0 to 1. The initial kinetic coefficient was defined as (eq 3):

3 $$k_{init}={\left(\frac{d\alpha }{dt}\right)}_{t\to 0}$$

The same initial time interval was used for adsorption and desorption at every temperature. Apparent activation energies were obtained from Arrhenius plots of ln [*k*_init_/(1 s^–1^)] against 1/*T*. An effective kinetic asymmetry factor and the associated energy-like descriptor were defined as (eq 4):

4 $$K_{kin}=\frac{k_{init,ads}}{k_{init,des}},\Delta G_{kin}=-\mathrm{RT}\;\ln K_{kin}$$

The temperature dependence was analyzed using eq 5:

5 $$\Delta G_{kin}\left(T\right)=\Delta H_{kin}-T\Delta S_{kin}$$

These quantities describe the effective local kinetic response detected by IR and are not treated as equilibrium adsorption thermodynamics. Full definitions and uncertainty propagation are given in the Supporting Information.

### 2.5 Evaporation Experiments

Bare CF and F-HIPF@CF evaporators were placed under a 50 W, 805 nm LED source. The incident optical power was varied from 0.085 to 0.76 W by changing the lamp–sample distance. Mass changes were recorded using an electronic balance with 0.1 mg resolution. The total evaporation flux, *J*(*P*), was obtained from the slope of the linear mass-loss trace normalized to the exposed area. Dark measurements were performed under otherwise equivalent conditions as a baseline control.

The directly observed effect of the immobilized HIPF phase was expressed as the relative increase in the total evaporation flux (eq 6):

6 $$R_{raw}\left(P\right)=[\frac{J_{F‐HIPF@CF}\left(P\right)}{J_{CF}\left(P\right)}-1]\times 100\%$$

## 3 Results and Discussion

### 3.1 Morphology, Crystallinity, and Spectroscopic Indications of Proton Transfer

The analysis SEM reveals two distinctly different morphologies (Figure 1A). F-HIPF consists of elongated fiber-like crystallites, whereas P-HIPF forms plate-like domains. The morphology-based prefixes distinguish the two growth habits without assigning framework dimensionality.

**Figure 1:**
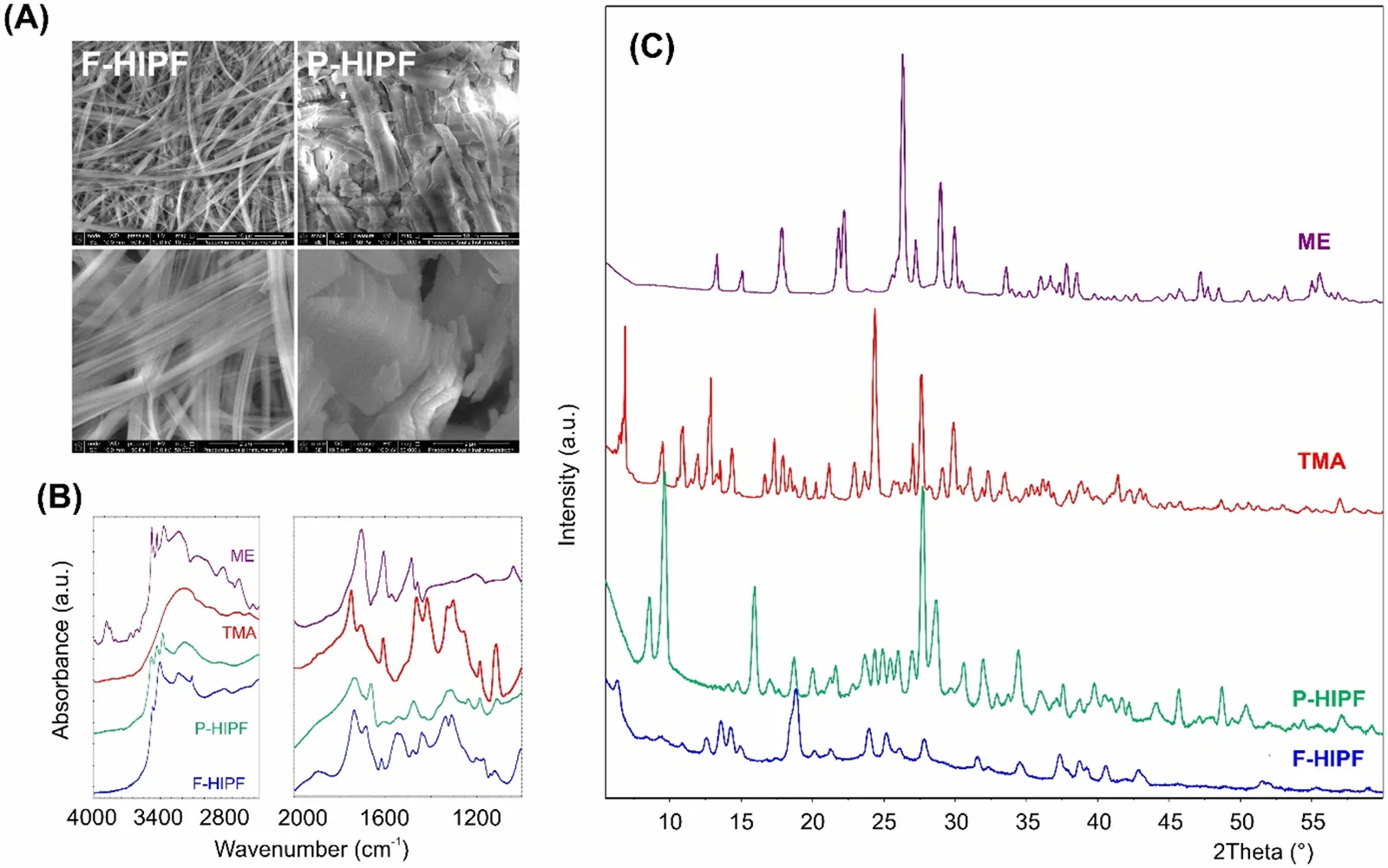
Structural and spectroscopic characterization of F-HIPF and P-HIPF. (A) SEM images showing the fiber-like morphology of F-HIPF and plate-like morphology of P-HIPF. (B) IR spectra of ME, TMA, F-HIPF, and P-HIPF. Bands near 1540 and 1440 cm^–1^ and an additional feature near 3115 cm^–1^ in F-HIPF support partial proton transfer and ion-pair formation. (C) PXRD patterns demonstrating that both HIPFs are crystalline phases distinct from ME and TMA.

The IR spectra of both products differ from those of the parent compounds (Figure 1B), demonstrating reorganization of the hydrogen-bonding environment. F-HIPF displays carboxylate-related features near 1540 and 1440 cm^–1^ and an additional band near 3115 cm^–1^. Together with the acid–base character of ME and TMA, these features support partial proton transfer and localized COO^‑^···HN^+^ interactions, providing the spectroscopic basis for classifying the materials as HIPFs. The precise spatial arrangement of the ionic motifs is considered in the proposed molecular model.

PXRD confirms that coprecipitation produces two crystalline phases with patterns distinct from those of ME and TMA (Figure 1C). P-HIPF exhibits a larger number of well-resolved reflections, while F-HIPF has a simpler pattern, demonstrating distinct crystal packing and long-range order in the two phases.

### 3.2 Low N_2_-Accessible Surface Area and Distinct Water-Vapor Responses

The N_2_ isotherms are nearly linear over most of the relative-pressure range and show no distinct low-pressure micropore-filling region. Their BET areas, 7.5 m^2^ g^–1^ for F-HIPF and 2.5 m^2^ g^–1^ for P-HIPF, characterize both materials as low-area and effectively nonporous to N_2_. A small uptake increase at high *p*/*p*_*s*_ is consistent with secondary spaces between particles (Figure S1). This low-porosity character is advantageous for establishing that the hydration response and subsequent evaporation effect do not require a developed permanent pore network.

The materials exhibit markedly different water-vapor isotherms (Figure 2A). P-HIPF displays pronounced adsorption–desorption hysteresis at high humidity, consistent with cooperative, condensation-like retention in secondary interparticle or interlamellar spaces. F-HIPF shows smooth, essentially hysteresis-free uptake and release, indicating a highly reversible hydration response.

**Figure 2:**
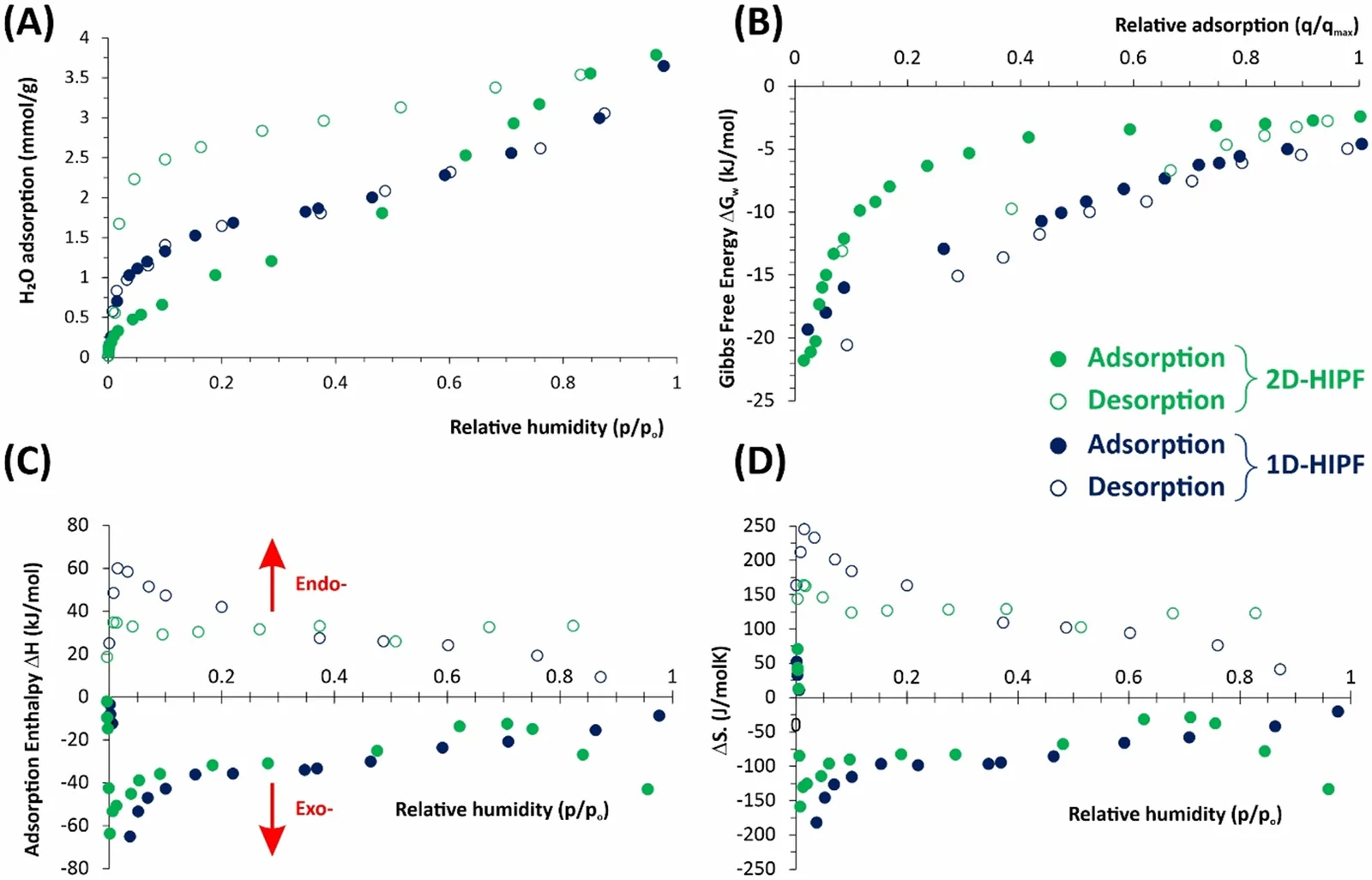
Water-vapor adsorption and branch-dependent thermodynamic signatures of F-HIPF and P-HIPF. (A) H_2_O adsorption–desorption isotherms at 25 °C. F-HIPF shows smooth, essentially hysteresis-free uptake, whereas P-HIPF exhibits pronounced high-humidity hysteresis. (B) The changes in water-vapor Gibbs free energies relative to saturation, mapped against fractional uptake, *q*/*q*_max_. (C) Directional differential molar enthalpy measured directly using differential Tian–Calvet calorimetry. (D) Corresponding direction-dependent entropy changes. Open and filled symbols denote the desorption and adsorption branches, respectively.

Mapping Δ*G*_w_ against *q*/*q*_max_ directly compares the hydration trajectories of the two HIPFs. At a given fractional uptake, F-HIPF and P-HIPF require different water activities, reflecting their distinct water–solid interactions and cooperative hydration behavior.

At low uptake, adsorption is strongly exothermic for both materials. With increasing uptake, F-HIPF becomes progressively less exothermic and reaches comparatively small magnitudes at high humidity. P-HIPF retains a substantially larger exothermic contribution at high uptake, consistent with stronger collective stabilization of the retained water population. The endothermic desorption branches reflect the heat required to release water from the corresponding hydrated states.

The adsorption-branch entropy changes are negative for both materials, particularly at low and intermediate uptake. The magnitude of the entropy loss becomes much smaller for F-HIPF at high uptake, whereas P-HIPF retains a larger negative contribution. F-HIPF is therefore characterized by weaker enthalpic stabilization and lower configurational restriction at high loading than P-HIPF.

### 3.3 Operando IR Response and Initial Kinetic Coefficients

Difference IR spectra collected during hydration and dehydration evolve smoothly and reversibly for both HIPFs (Figures 3A and 4A). F-HIPF primarily changes in intensity with limited evolution of the O–H envelope, whereas P-HIPF develops broader features near 3250–3350 and approximately 3000 cm^–1^. These observations indicate a greater contribution from correlated hydrogen-bonded water populations in P-HIPF and a more uniform distribution of local water environments in F-HIPF.

**Figure 3:**
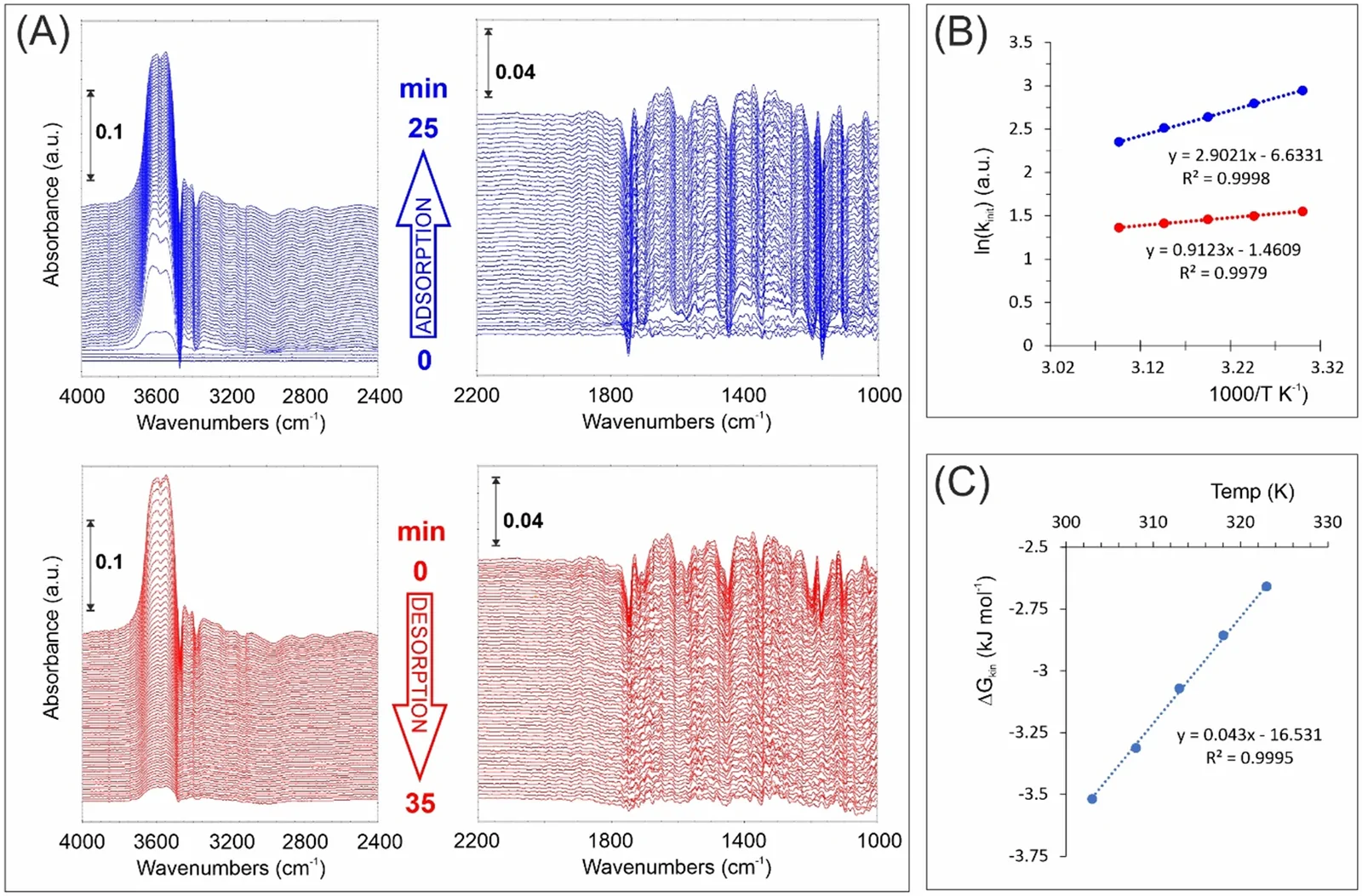
Operando IR response and kinetic descriptors for F-HIPF. (A) Time-resolved difference IR spectra during adsorption (blue) and desorption (red). (B) Arrhenius plots of the normalized initial kinetic coefficients, ln [*k*_init_/(1 s^–1^)], yielding *E*_*a*,ads_^app^ = −24.13 kJ mol^–1^ and *E*_*a*,des_^app^ = −7.59 kJ mol^–1^. (C) Temperature dependence of Δ*G*_kin_ = −*RT* ln (*k*_init,ads_/*k*_init,des_). Linear analysis gives Δ*H*_kin_ = −16.53 kJ mol^–1^ and Δ*S*_kin_ = −43.0 J mol^–1^ K^–1^. These are effective kinetic descriptors of the IR-observed response.

**Figure 4:**
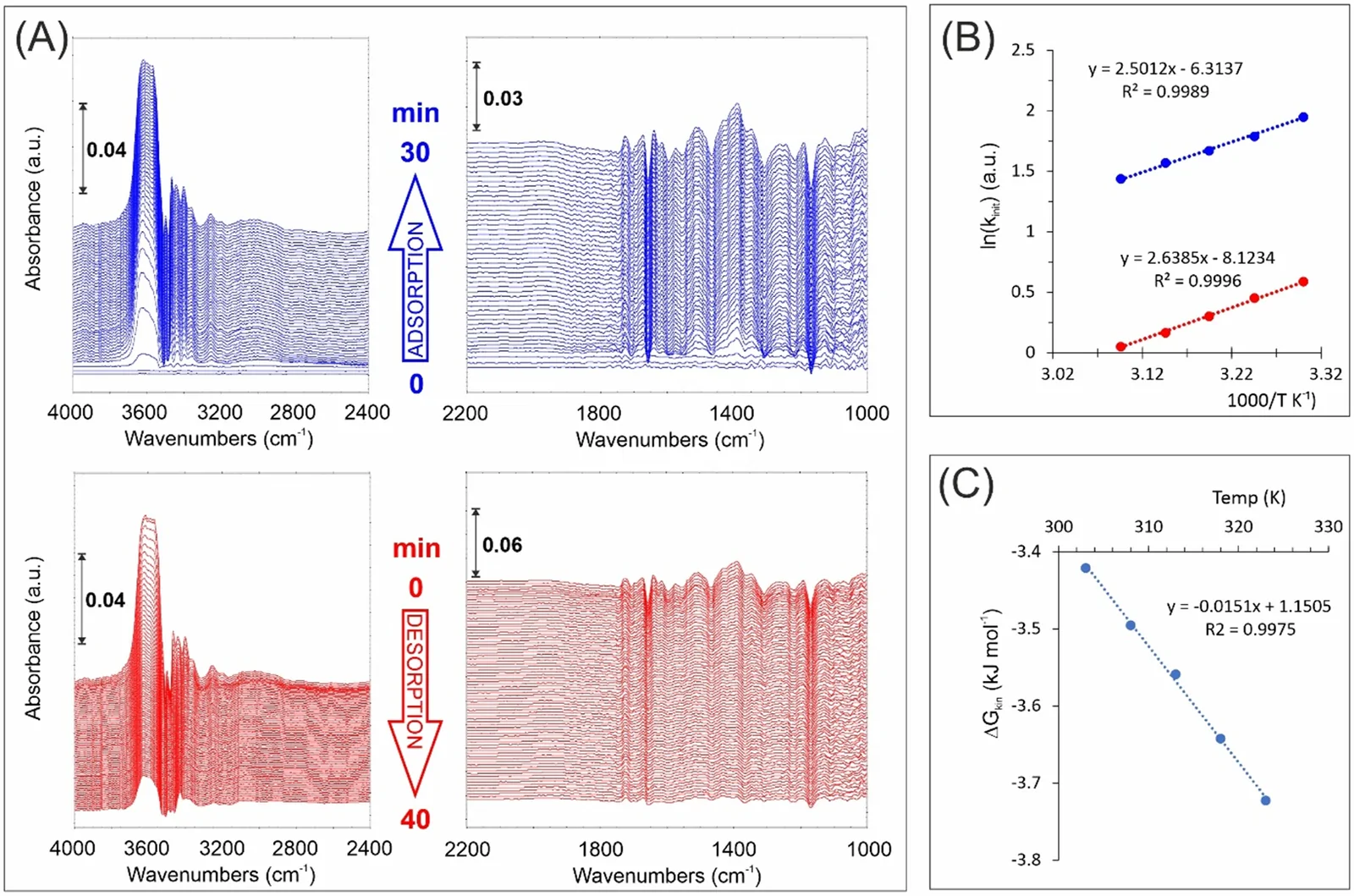
Operando IR response and kinetic descriptors for P-HIPF. (A) Time-resolved difference IR spectra during adsorption (blue) and desorption (red). (B) Arrhenius plots of *k*_init_, yielding *E*_*a*,ads_^app^ = −20.80 kJ mol^–1^ and *E*_*a*,des_^app^ = −21.94 kJ mol^–1^. (C) Temperature dependence of Δ*G*_kin_, giving Δ*H*_kin_ = +1.15 kJ mol^–1^ and Δ*S*_kin_ = +15.1 J mol^–1^ K^–1^.

For F-HIPF, both *k*_init,ads_ and *k*_init,des_ decrease with increasing temperature, giving negative apparent activation energies of approximately −24.13 and −7.59 kJ mol^–1^, respectively (Figure 3B). Negative apparent values indicate that the normalized initial response is governed by coupled temperature-dependent population, adsorption-driving-force, and reorganization effects rather than by a single elementary barrier.

The greater temperature sensitivity of adsorption than desorption in F-HIPF results in Δ*H*_kin_ = −16.53 kJ mol^–1^ and Δ*S*_kin_ = −43.0 J mol^–1^ K^–1^. Thus, the local response monitored by IR is enthalpically favorable but accompanied by an entropic penalty. The descriptor becomes less negative with increasing temperature, consistent with progressive weakening of the kinetic preference for uptake.

For P-HIPF, the apparent activation energies are similar: *E*_*a*,ads_^app^ = −20.80 kJ mol^–1^ and *E*_*a*,des_^app^ = −21.94 kJ mol^–1^ (Figure 4B). Their difference is small, giving Δ*H*_kin_ = +1.15 kJ mol^–1^, while Δ*S*_kin_ = +15.1 J mol^–1^ K^–1^. The P-HIPF response is therefore almost athermal and kinetically entropy-favored over the investigated range.

It is worth noting that Figure 2 depicts the equilibrium hydration trajectory using water activity and direct calorimetric data, integrating coupled water–surface, water–water, and structural contributions. Figures 3 and 4 describe the initial normalized response of selected IR bands after a change in water-vapor conditions. Together, these measurements provide complementary equilibrium thermodynamic and effective kinetic descriptions of hydration.

### 3.4 Irradiation-Enhanced Evaporation on Coated Carbon Fibers

Among the two HIPFs, only F-HIPF exhibits essentially hysteresis-free water adsorption–desorption. It was therefore selected for immobilization on carbon fibers because continuous evaporation requires repeated water uptake, transport, and release. P-HIPF, which retains water through a pronounced high-humidity hysteresis loop, was not examined as an evaporation coating.

The SEM analysis confirms that F-HIPF forms a dense crystalline coating on oxidized carbon fibers (Figure 5A,B). Under 805 nm irradiation, both bare CF and F-HIPF@CF display approximately linear mass loss with time, while the coated material shows a higher evaporation flux throughout the investigated power range (Figure 5C).

**Figure 5:**
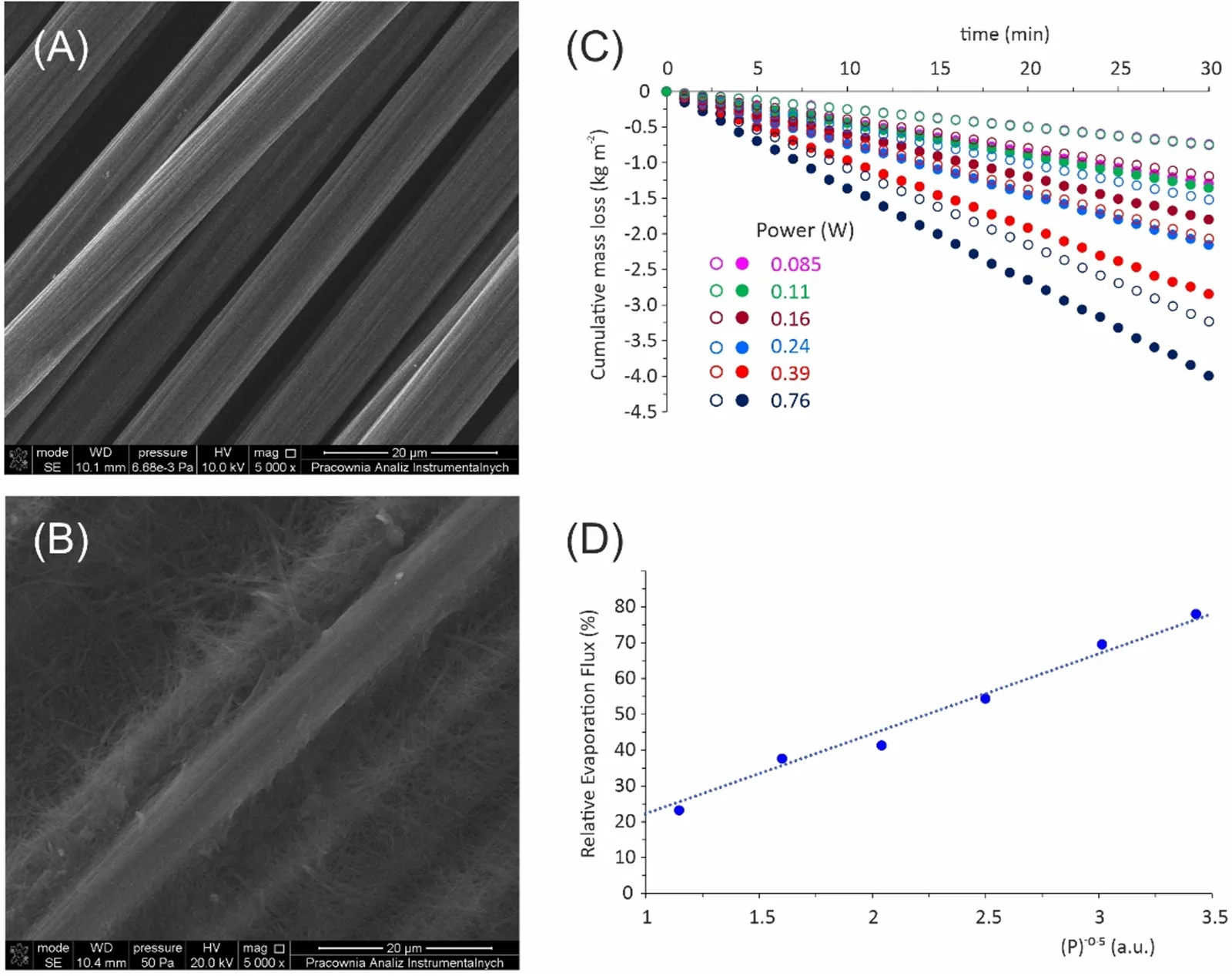
Morphology and evaporation response of bare CF and F-HIPF@CF. (A) Bare carbon fibers. (B) F-HIPF crystallites immobilized on CF. (C) Cumulative mass loss per unit area under 805 nm irradiation at incident powers of 0.085–0.76 W; filled and open symbols denote F-HIPF@CF and bare CF, respectively. The slopes of the linear traces give the total evaporation flux, *J*(*P*). (D) Relative increase in the total evaporation flux produced by the immobilized F-HIPF phase, *R*_raw_ = [*J*_F–HIPF@CF_/*J*_CF_ – 1] × 100%. The fitted trend is empirical and is limited to the investigated power range.

Figure 5D reports the directly observed relative effect of adding F-HIPF to the CF support. Across the investigated power range, F-HIPF@CF exhibits an approximately 23–78% higher total evaporation flux than bare CF. In the absence of irradiation, bare CF and F-HIPF@CF show comparable fluxes of approximately 700 and 720 g m^–2^ h^–1^, respectively. The small difference under dark conditions confirms that immobilization does not itself produce a substantial increase in baseline evaporation; the larger relative effect develops under irradiation.

The evaporation response arises within a three-dimensional fibrous network containing directly illuminated external fibers, shaded internal fibers, wet pathways, and spatially distributed evaporation zones. The observed enhancement is therefore consistent with coupled energy deposition, capillary water replenishment, vapor transport, and the reversible hydration of the immobilized F-HIPF phase. The present measurements establish the net functional contribution of F-HIPF under the tested irradiation conditions, while the relative roles of the individual transport and thermal contributions will be considered separately.

### 3.5 Proposed Molecular Interpretation

The combined results support a morphology-dependent difference in hydration response. A working molecular model proposes that the hot-mixing/slow-cooling route favors a greater contribution from ion-paired ME–TMA motifs and anisotropic growth, whereas cold mixing produces a distinct packing arrangement with a greater tendency toward plate-like crystallization. This interpretation connects the IR, PXRD, and morphological observations with the contrasting hydration behavior, as summarized schematically in Figure 6.

**Figure 6:**
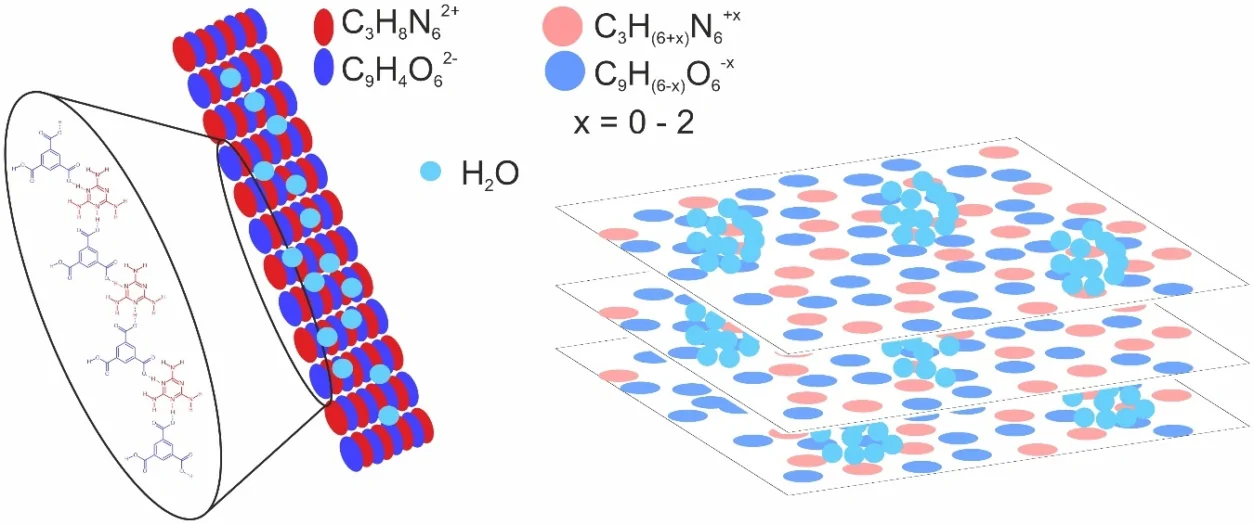
Proposed molecular interpretation of F-HIPF and P-HIPF. The schematic illustrates differences in proton transfer, packing, and hydration organization proposed for the fiber-like and plate-like phases. The model connects the ion-paired ME–TMA motifs with the distinct water populations indicated by calorimetry, water-vapor adsorption, and operando IR spectroscopy.

F-HIPF combines low N_2_-accessible surface area, a nearly reversible water isotherm, a smaller high-humidity enthalpic and entropic change, and limited spectral evolution of the O–H band. Collectively, these observations are consistent with a reduced tendency toward strongly cooperative water organization. P-HIPF is also nonporous to N_2_ but develops pronounced water hysteresis and broader O–H features, supporting more cooperative, condensation-like retention in secondary spaces.

The evaporation experiment extends this comparison to an operating three-dimensional interface. Similar dark evaporation establishes a common baseline for bare and coated fibers, whereas the 23–78% increase under irradiation demonstrates the functional contribution of F-HIPF. Its reversible hydration provides a molecularly responsive component coupled to heat and mass transport across the fibrous network.

## 4 Conclusions

Changing the nucleation protocol of the same ME and TMA precursors produces two crystalline HIPF phases with fiber-like and plate-like morphologies. SEM and PXRD establish distinct growth habits and crystalline packing, while IR spectroscopy supports proton-transferred, ion-paired motifs characteristic of HIPFs.

Both HIPFs have low N_2_-accessible surface areas and minimal permanent microporosity, demonstrating that a developed pore network is not required for their interaction with water. F-HIPF exhibits smooth, hysteresis-free uptake and a reduced high-humidity enthalpic and entropic change. P-HIPF develops pronounced hysteresis and spectral signatures consistent with more cooperative, condensation-like water retention in secondary interparticle or interlamellar spaces.

The normalized initial IR responses provide a complementary kinetic description. F-HIPF displays an enthalpically favorable but entropically penalized kinetic response, whereas P-HIPF is nearly athermal and entropy-favored. These effective kinetic descriptors are distinct from equilibrium or net hydration thermodynamics.

F-HIPF was selected for immobilization because its essentially hysteresis-free hydration is compatible with repeated water uptake and release. When deposited on oxidized carbon fibers, it increases the total evaporation flux by approximately 23–78% relative to bare CF across the investigated irradiation range, while dark evaporation remains comparable. The results demonstrate that nucleation-controlled HIPF formation can alter hydration and evaporation performance even when permanent N_2_-accessible porosity is minimal.

## Supplementary Material



## Data Availability

Data supporting the findings are available within the article and Supporting Information. Additional raw adsorption, spectroscopic, and evaporation datasets are available from the corresponding author upon reasonable request.

## Notes

Low temperature N_2_ adsorption isotherms and BET surface area values; water-vapor adsorption protocol; differential Tian–Calvet calorimetry and thermodynamic quantities determinations; normalization of operando IR responses and determination of *k*_init_; effective kinetic asymmetry descriptors; and relative evaporation response and dark control (PDF)
