# Supplementary material for: Suppressing Cooperative Water Condensation through Nucleation-Controlled HIPF Architectures
Source: Langmuir. 2026 Sep 17;42(38):28193–200. doi: 10.1021/acs.langmuir.6c03562 (PMC13629667; doi:10.1021/acs.langmuir.6c03562)
Supplement: Supplementary file 1 [file la6c03562_si_001.pdf]

Supporting Information

Suppressing Cooperative Water Condensation through Nucleation-Controlled HIPF Architectures

Marek Wiśniewski*

1) Department of Materials Chemistry, Adsorption and Catalysis, Faculty of Chemistry, Nicolaus Copernicus University in Toruń, Gagarina 7, 87-100 Toruń, Poland

2) Institute of Advanced Studies, Nicolaus Copernicus University in Toruń, Wileńska 4, 87-100 Toruń, Poland

email address: marekw@umk.pl

S1. Nitrogen adsorption and BET surface area

The low temperature N_2 adsorption–desorption isotherms were measured at 77 K. Both ME–TMA HIPFs showed nearly linear uptake over most of the relative-pressure range, without a distinct low-p/p_s micropore-filling region. A modest increase in uptake at high p/p_s is attributed to secondary spaces between particles or crystallite aggregates. The isotherm shapes and low BET areas characterize both materials as low-area and effectively nonporous to N_2. This low-porosity platform allows the hydration and evaporation responses to be evaluated without a dominant contribution from extensive permanent-pore filling.

Table S1. N_2-accessible BET surface areas of F-HIPF and P-HIPF.

Sample	Morphological description	S_{BET} (m² g⁻¹)	Interpretation
F-HIPF	Fiber-like HIPF	7.5	Low-area, effectively nonporous to N_2
P-HIPF	Plate-like HIPF	2.5	Low-area, effectively nonporous to N_2

The small surface areas make a quantitative pore-size distribution physically unreliable. Accordingly, no intrinsic pore-size distribution is reported. The high-humidity hysteresis observed for P-HIPF in the water-vapor experiment is therefore discussed as cooperative, condensation-like retention potentially involving interparticle or interlamellar spaces rather than capillary condensation in a permanent N_2-accessible framework pore network.

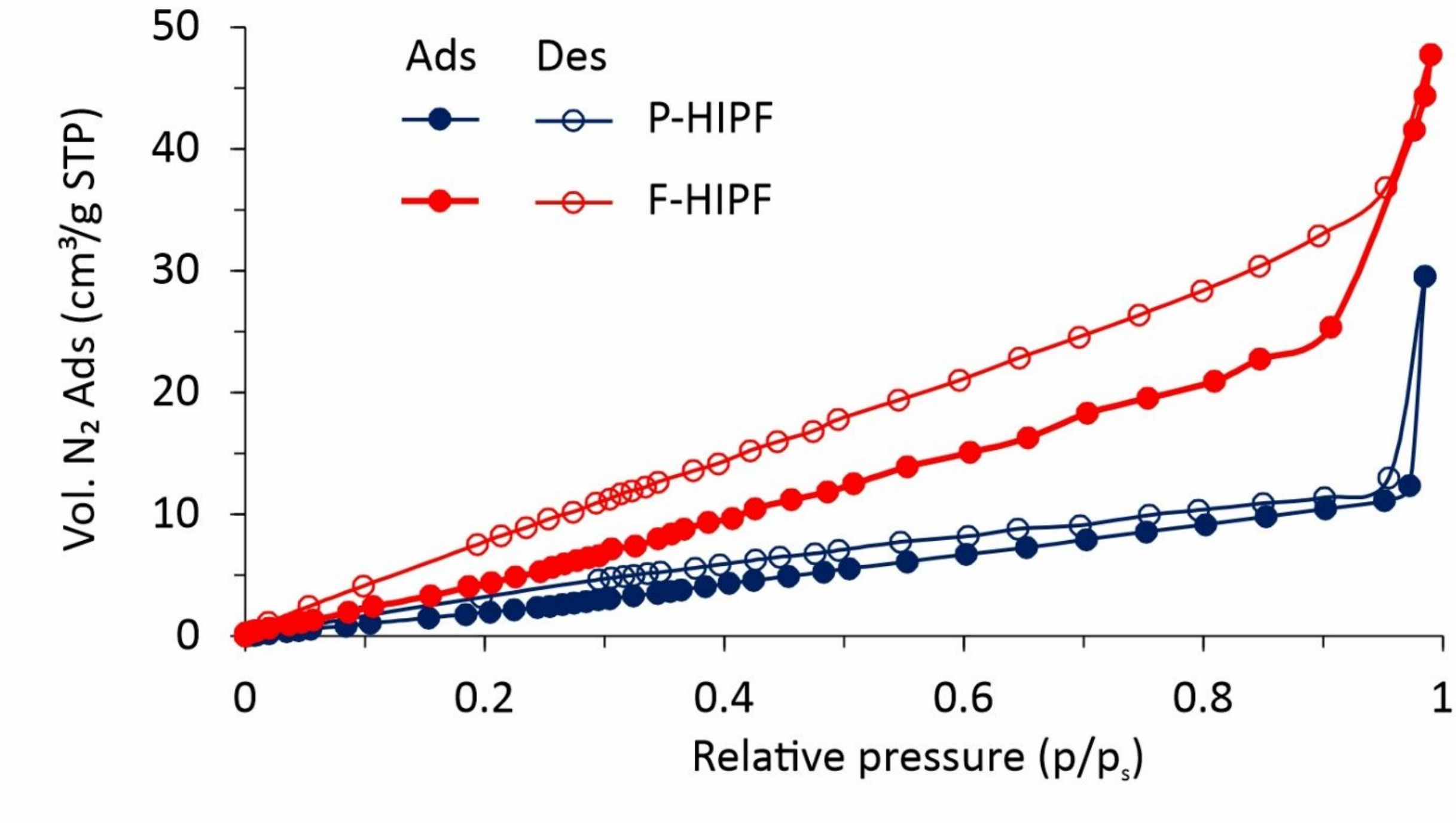

Figure S1. N_2 adsorption–desorption isotherms of F-HIPF and P-HIPF at 77 K. The nearly linear low-uptake isotherms and low BET areas indicate minimal N_2-accessible permanent porosity. The modest increase at high p/p_s is assigned to secondary interparticle voids.

S2. Water-vapor adsorption protocol

Before water-vapor adsorption, the samples were evacuated at 50 °C until constant mass was obtained. The principal adsorption–desorption isotherms were measured at 25 °C. Pressure was continuously monitored after every dose, and each point was allowed to equilibrate for at least 5 h.

The water activity was calculated as

$$a_{\mathrm{w}} = \frac{p}{p_s(T)},$$

where p is the measured water-vapor pressure and $p_s(T)$ is the saturation pressure at the measurement temperature. The chemical-potential difference relative to saturated water vapor at the same temperature was calculated under the ideal-vapor approximation as

$$\Delta G_{\mathrm{w}} = \Delta\mu_{\mathrm{w}} = RT\ln a_{\mathrm{w}} = RT\ln\left(\frac{p}{p_s}\right).$$

Because $\Delta\mu_{\mathrm{w}}$ is imposed by water activity, it is material-independent as a function of p/p_s. To compare the hydration trajectories of the two HIPFs, it was mapped against the normalized uptake

$$\theta = \frac{q}{q_{\max}},$$

where $q_{\max}$ is the maximum uptake measured for the corresponding sample and branch within the investigated range.

All measurements were performed in triplicate. The reported values represent the mean of three repeat measurements, and the relative standard deviation did not exceed 3%.

S3. Differential Tian–Calvet calorimetry and thermodynamic quantities

Water adsorption and desorption heats were measured directly using a differential Tian–Calvet-type adsorption calorimeter equipped with two geometrically identical cells operated as sample and reference channels. Both cells were subjected to the same water-vapor pressure program. The differential arrangement largely compensated for common thermal contributions arising from gas expansion or compression, transport through the dosing system, and pressure-step perturbations. The remaining differential heat-flow signal was integrated for each pressure step and normalized to the amount of water adsorbed or desorbed during that step.

Directional differential molar enthalpies were reported using the experimentally observed process convention:

$$\Delta H_{\mathrm{ads}} < 0 \quad \text{(exothermic adsorption)}, \qquad \Delta H_{\mathrm{des}} > 0 \quad \text{(endothermic desorption)}.$$

The equilibrium water chemical potential relative to saturated water vapor was calculated for both branches as

$$\Delta\mu_{\mathrm{w}} = RT\ln(p/p_s).$$

This quantity becomes progressively more negative as water-vapor pressure decreases. During a pressure-reduction step, desorption proceeds because the chemical potential of the new gas reservoir is lower than that of the initially hydrated material. At the end of each step, the material and gas phase reach a new equilibrium.

The direction-dependent entropy changes shown in Figure 2D were calculated as

$$\Delta S_{\mathrm{ads}} = \frac{\Delta H_{\mathrm{ads}} - \Delta\mu_{\mathrm{w}}}{T},$$

$$\Delta S_{\mathrm{des}} = \frac{\Delta H_{\mathrm{des}} + \Delta\mu_{\mathrm{w}}}{T}.$$

The second expression accounts for the reversed direction of the desorption process while retaining $\Delta\mu_{\mathrm{w}}$ in Figure 2B as the equilibrium chemical potential relative to saturation.

S4. Normalization of operando IR responses and determination of k_{init}

Time-resolved IR spectra were recorded as difference spectra relative to the initial state. Therefore, the initial difference-spectrum signal is $A_0 = 0$. For adsorption, the normalized progress variable was defined as

$$\alpha_{\mathrm{ads}}(t) = \frac{A(t) - A_0}{A_\infty - A_0} = \frac{A(t)}{A_\infty}.$$

For desorption, the sign was chosen so that the progress variable also increased from 0 to 1:

$$\alpha_{\mathrm{des}}(t) = \frac{A_0 - A(t)}{A_0 - A_\infty},$$

where A_0 and A_∞ refer to the initial hydrated and final dehydrated difference-spectrum signals for the desorption step. Equivalently, the absolute normalized signal change may be used, provided that the same convention is applied to all temperatures.

The initial kinetic coefficient was determined by linear regression of the common initial time interval:

$$\alpha_i(t) = k_{\mathrm{init},i}\,t,$$

$$k_{\mathrm{init},i} = \left(\frac{d\alpha_i}{dt}\right)_{t\to 0}, \qquad [k_{\mathrm{init}}] = \mathrm{s}^{-1}.$$

Because α is dimensionless, k_{init} is independent of the absolute absorbance scale. The Arrhenius plots were expressed in dimensionless logarithmic form:

$$\ln\left[\frac{k_{\text{init}}}{1\ \text{s}^{-1}}\right] = \ln C - \frac{E_a^{\text{app}}}{RT}.$$

The resulting E_a^{app} values are described as apparent because the initial response can include changes in population, adsorption driving force, and interfacial reorganization in addition to an elementary kinetic barrier.

S5. Effective kinetic asymmetry descriptors

The dimensionless kinetic asymmetry factor was defined as

$$K_{\text{kin}}(T) = \frac{k_{\text{init,ads}}(T)}{k_{\text{init,des}}(T)}.$$

The associated energy-scaled descriptor was calculated as

$$\Delta G_{\text{kin}}(T) = -RT\ln K_{\text{kin}}(T).$$

Its temperature dependence was analyzed using

$$\Delta G_{\text{kin}}(T) = \Delta H_{\text{kin}} - T\Delta S_{\text{kin}}.$$

Thus, the intercept of ΔG_{kin} versus T gives ΔH_{kin}, and the slope gives $-\Delta S_{\text{kin}}$. These are effective descriptors of the asymmetry between the normalized initial adsorption and desorption responses. K_{kin} is not an equilibrium constant, and ΔG_{kin}, ΔH_{kin}, and ΔS_{kin} are not standard-state equilibrium adsorption functions.

Table S2. Effective parameters derived from the normalized initial IR response.

Sample	$E_{a,\text{ads}}^{\text{app}}$ (kJ mol⁻¹)	$E_{a,\text{des}}^{\text{app}}$ (kJ mol⁻¹)	ΔH_{kin} (kJ mol⁻¹)	ΔS_{kin} (J mol⁻¹ K⁻¹)
F-HIPF	−24.13	−7.59	−16.53	−43.0
P-HIPF	−20.80	−21.94	+1.15	+15.1

For F-HIPF,

$$E_{a,\text{ads}}^{\text{app}} - E_{a,\text{des}}^{\text{app}} = -16.54\ \text{kJ mol}^{-1},$$

which agrees with $\Delta H_{\text{kin}} = -16.53$ kJ mol⁻¹ obtained from the $\Delta G_{\text{kin}}(T)$ intercept. For P-HIPF, the corresponding difference is approximately +1.14 kJ mol⁻¹, consistent with $\Delta H_{\text{kin}} = +1.15$ kJ mol⁻¹. The algebraic agreement confirms the internal consistency of the kinetic analysis.

All measurements were performed in triplicate. The reported values represent the mean of three repeat measurements, and the relative standard deviation did not exceed 2%. Error bars represent the standard deviation of three repeat measurements. Where not visible, they are smaller than the symbol size.

S6. Relative evaporation response and dark control

The time-dependent quantity plotted in Figure 5C is the cumulative mass loss normalized to the exposed area, $\Delta m/A$. The total evaporation flux was obtained from the magnitude of the linear slope:

$$J(P) = -\frac{d(\Delta m/A)}{dt}.$$

The net functional effect of immobilizing F-HIPF on the carbon-fiber support was expressed as the directly observed relative increase in the total evaporation flux:

$$R_{\mathrm{raw}}(P) = \left[\frac{J_{\mathrm{F-HIPF@CF}}(P)}{J_{\mathrm{CF}}(P)} - 1\right] \times 100\%.$$

Across the investigated power range, R_{raw} increases from approximately 23% to 78%. These values quantify the total effect of the additional F-HIPF phase under otherwise equivalent irradiation conditions.

The dark evaporation fluxes of bare CF and F-HIPF@CF were approximately 700 and 720 g m^{-2} h^{-1}, respectively. Their close agreement shows that immobilization does not produce a substantial increase in baseline evaporation without irradiation. The dark measurements are therefore used as a control rather than subtracted from the values reported in Figure 5D.

Error bars represent the standard deviation of three repeat measurements. Where not visible, they are smaller than the symbol size
